# PERCEIVED RISK OF ALZHEIMER’S DISEASE/DEMENTIAS AND COGNITIVE FUNCTION AMONG US OLDER ADULTS

**DOI:** 10.1093/geroni/igad104.0659

**Published:** 2023-12-21

**Authors:** Nan Wang, Hanzhang Xu, Jessica West, Truls Østbye, Bei Wu, Ying Xian, Matthew Dupre

**Affiliations:** University of California, Davis, Davis, California, United States; Duke University, Durham, North Carolina, United States; Duke University, Durham, North Carolina, United States; Duke University School of Medicine, Durham, North Carolina, United States; New York University, New York City, New York, United States; UT Southwestern Medical Center, Dallas, Texas, United States; Duke University, Durham, North Carolina, United States

## Abstract

To assess factors associated with the perceived risk of Alzheimer’s disease and related dementias (ADRD) and how the perceived risk of ADRD was related to cognitive function. We conducted a retrospective cohort study using 5 waves of data from the Health and Retirement Study (2012-2022) that included adults aged 65 years or older with no previous diagnosis of ADRD at baseline. Cognitive function was measured at baseline and over time using a summary score that included immediate/delayed word recall, serial 7’s test, objective naming test, backwards counting, recall of the current date, and naming the president/vice-president (range=0-35). Perceived risk of developing ADRD was categorized at baseline as “definitely not” (0% probability), “unlikely” (1-49%), “uncertain” (50%), and “more than likely” (>50-100%). Additional baseline measures included participants’ sociodemographic background, psychosocial resources, health behaviors, physiological status, and healthcare utilization. Of 1,457 participants (median age 74 [IQR=63-85] and 59.8% women), individuals who perceived that they would “definitely not” develop ADRD were more likely to be non-Hispanic Black, less educated, and have lower household income than individuals who indicated it was “unlikely” they would develop ADRD. Participants who reported their risks of developing ADRD as “definitely not,” “uncertain,” and “more than likely” had lower cognitive function than participants who perceived their risk of developing ADRD as “unlikely.” Perceived risk of developing ADRD is associated with cognitive function. The (dis)concordance between individuals’ perceived risk of ADRD and their cognitive function has implications for increasing public awareness and developing interventions to prevent ADRD.

